# Transcriptional Control of Cell Lineage Development in Epicardium-Derived Cells

**DOI:** 10.3390/jdb1020092

**Published:** 2013-07-03

**Authors:** Caitlin M. Braitsch, Katherine E. Yutzey

**Affiliations:** The Heart Institute, Division of Molecular Cardiovascular Biology, Cincinnati Children’ Hospital, Medical Center, 240 Albert Sabin Way ML 7020, Cincinnati, OH 45229, USA

**Keywords:** transcription factor, Wt1, Tcf21, Tbx18, epicardium derived cell, embryo, cardiovascular disease

## Abstract

Epicardial derivatives, including vascular smooth muscle cells and cardiac fibroblasts, are crucial for proper development of the coronary vasculature and cardiac fibrous matrix, both of which support myocardial integrity and function in the normal heart. Epicardial formation, epithelial-to-mesenchymal transition (EMT), and epicardium-derived cell (EPDC) differentiation are precisely regulated by complex interactions among signaling molecules and transcription factors. Here we review the roles of critical transcription factors that are required for specific aspects of epicardial development, EMT, and EPDC lineage specification in development and disease. Epicardial cells and subepicardial EPDCs express transcription factors including Wt1, Tcf21, Tbx18, and Nfatc1. As EPDCs invade the myocardium, epicardial progenitor transcription factors such as Wt1 are downregulated. EPDC differentiation into SMC and fibroblast lineages is precisely regulated by a complex network of transcription factors, including Tcf21 and Tbx18. These and other transcription factors also regulate epicardial EMT, EPDC invasion, and lineage maturation. In addition, there is increasing evidence that epicardial transcription factors are reactivated with adult cardiac ischemic injury. Determining the function of reactivated epicardial cells in myocardial infarction and fibrosis may improve our understanding of the pathogenesis of heart disease.

## 1. Epicardium-Derived Cells (EPDCs) in Heart Development and Disease

In the developing heart, cells that form the coronary vessels and the cardiac fibrous matrix are derived from the epicardium and are required for cardiac function [1,2]. Specifically, epicardium-derived cells (EPDCs), generated from the epicardial cell layer by an epithelial-to-mesenchymal transition (EMT), include progenitors of coronary vascular smooth muscle cells (SMCs) and cardiac fibroblasts. Additional Cre-based lineage analysis and cell fate mapping studies provide evidence that EPDCs contribute to vascular endothelial cell and cardiomyocyte lineages [3–7]. Several transcription factors including Wt1, Tcf21, Tbx18, and Nfatc1 have been implicated in epicardial EMT and EPDC lineage development [8–13]. Congenital abnormalities in EPDC lineages can lead to coronary artery anomalies that occur in 1.3% of the population often resulting in life-threatening arrhythmia, myocardial infarction (MI), or even sudden death [14]. Likewise, epicardium-derived coronary vascular SMCs and cardiac fibroblasts may be reactivated in adult heart disease and cardiac fibrosis [4,15]. Following MI, the epicardium is activated, with new EPDC formation and epicardial transcription factor reactivation suggesting a potential role in adult cardiac injury response, fibrosis, and pathology [16–18]. Thus, there is increasing evidence for recapitulation of epicardial transcriptional developmental regulatory mechanisms in adult cardiovascular disease.

## 2. Overview of Epicardial Formation and Cell Lineage Diversification

In the vertebrate embryo, the proepicardium (PE) is derived from the splanchnic mesoderm and forms as a cluster of mesothelial cells located between the liver and cardiac sinus venosus [19]. Although transient, the PE is significant in that it contributes multiple cell lineages required for heart function, including fibroblasts and coronary smooth muscle (SM) [6,20]. As the primitive heart loops to form the four-chambered heart, the cells of the PE, located at the venous pole of the heart, proliferate and migrate over the myocardium to form the epithelial epicardium [21]. A subset of epicardial cells undergoes EMT and invades the subepicardial space and then the myocardium [20]. Epicardial EMT is evident by embryonic day 11.5 (E11.5) in mice and E3 in chick [22,23]. Following invasion into the myocardium, the majority of progenitor EPDCs differentiates into vascular SMCs, adventitial fibroblasts that support the coronary vasculature, or interstitial fibroblasts that generate the cardiac fibrous matrix [4,7,19,24].

Early retroviral labeling lineage studies and quail-chick chimera experiments indicated that EPDCs contribute to fibroblast, SM, and coronary endothelial cell lineages [1,2]. Subsequent Cre-based fate mapping experiments confirmed epicardial origin of these lineages and also suggested that epicardial derivatives may contribute to cardiomyocytes [3,6,7]. However, the extent to which EPDCs differentiate into coronary endothelium and cardiomyocytes is controversial [3,5–7,25–27]. In the atrioventricular (AV) sulcus, subepicardial mesenchymal cells coalesce to form a primitive capillary plexus, which later remodels to form the mature coronary vasculature [28], and also contributes to the fibrous annulus and parietal AV valve leaflets [24,29,30]. While it is known that multiple cell types arise from epicardial progenitors, the timing and regulation of SMC, fibroblast, and endothelial cell lineage determination is not fully characterized. In addition it is not known if the various epicardial derivatives arise from common or distinct progenitor pools. Recent studies provide evidence that EPDC lineages arise from distinct populations located at the venous pole of the heart and are specified prior to epicardial EMT [6,13].

## 3. Transcriptional Regulation of Epicardial EMT and EPDC Lineage Specification

**Figure 1:**
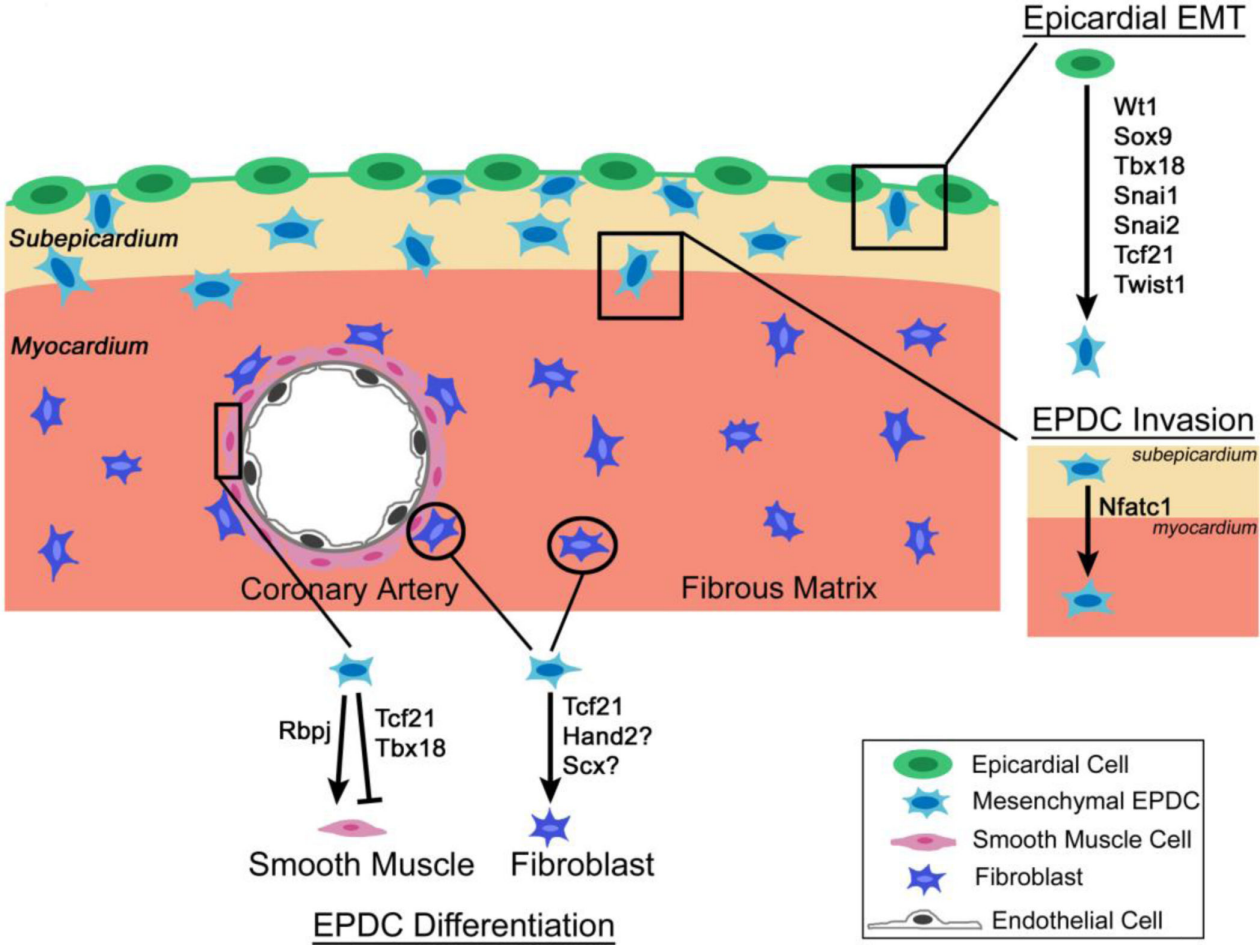
Schematic depicting transcription factor regulation of epicardial cells during embryonic heart development. Several transcription factors are expressed during epicardial epithelial-to-mesenchymal transition (EMT), epicardium-derived cell (EPDC) lineage specification, and EPDC differentiation into vascular smooth muscle cells and cardiac fibroblasts. See text for details and references.

Initial formation of the epicardium, epicardial EMT, and EPDC lineage determination are regulated by a complex network of transcription factors, including the zinc finger transcription factors Wt1, Snai1, and Snai2, as well as the bHLH transcription factors Tcf21, Scleraxis, Twist1, and Hand2 (Figure 1) [8–11]. Additional factors, including Tbx18, Nfatc1, Sox9, and C/EBP, regulate aspects of EPDC lineage development. Signaling pathways and transcription factors together regulate EPDC behavior and differentiation into cardiac fibroblasts and vascular SMCs [4]. Transcription factors expressed in EPDCs, including Wt1, Tbx18, Tcf21, Snai1, and C/EBP, are reactivated in cardiac injury and may mark progenitor or reparative populations in the disease state [18,31,32].

### 3.1. Wt1

The zinc finger transcription factor Wt1 was originally described as a tumor suppressor gene that is mutated in Wilms’ tumor patients [33]. Wt1 is robustly expressed in the septum transversum/ pericardial mesothelium, the PE, and the epicardium [34,35]. Following epicardial EMT, Wt1 expression is rapidly downregulated in invading EPDCs in the developing heart [36]. Therefore, Wt1 is expressed in EPDC progenitors with expression that diminishes prior to EPDC differentiation. Mice lacking Wt1 have epicardial defects with a paucity of EPDCs, suggesting an EMT defect [12,37,38]. Wt1 is necessary and sufficient to activate transcription of *α4integrin (Itga4)* via the proximal promoter (Table 1), and *Itga4* is required to maintain epicardial adhesion and integrity [11]. In addition, Wt1 directly regulates *Snai1* and *Snai2 (Slug)* transcription in the epicardium [39,40]. Therefore, Wt1 is a crucial component of the mechanism regulating epicardial adhesion and EMT. Wt1 is required to promote epicardial expression of additional downstream targets, including *Nestin*, a component of intermediate filaments, *TrkB* (Tyrosine kinase type B receptor), important for BDNF (brain-derived neurotrophic factor) signaling and vascularization, and *Coronin1B*, which is crucial for cell motility [41–43]. Thus, loss of Wt1 adversely affects the cytoskeleton, thereby impacting EMT.

**Figure 2:**
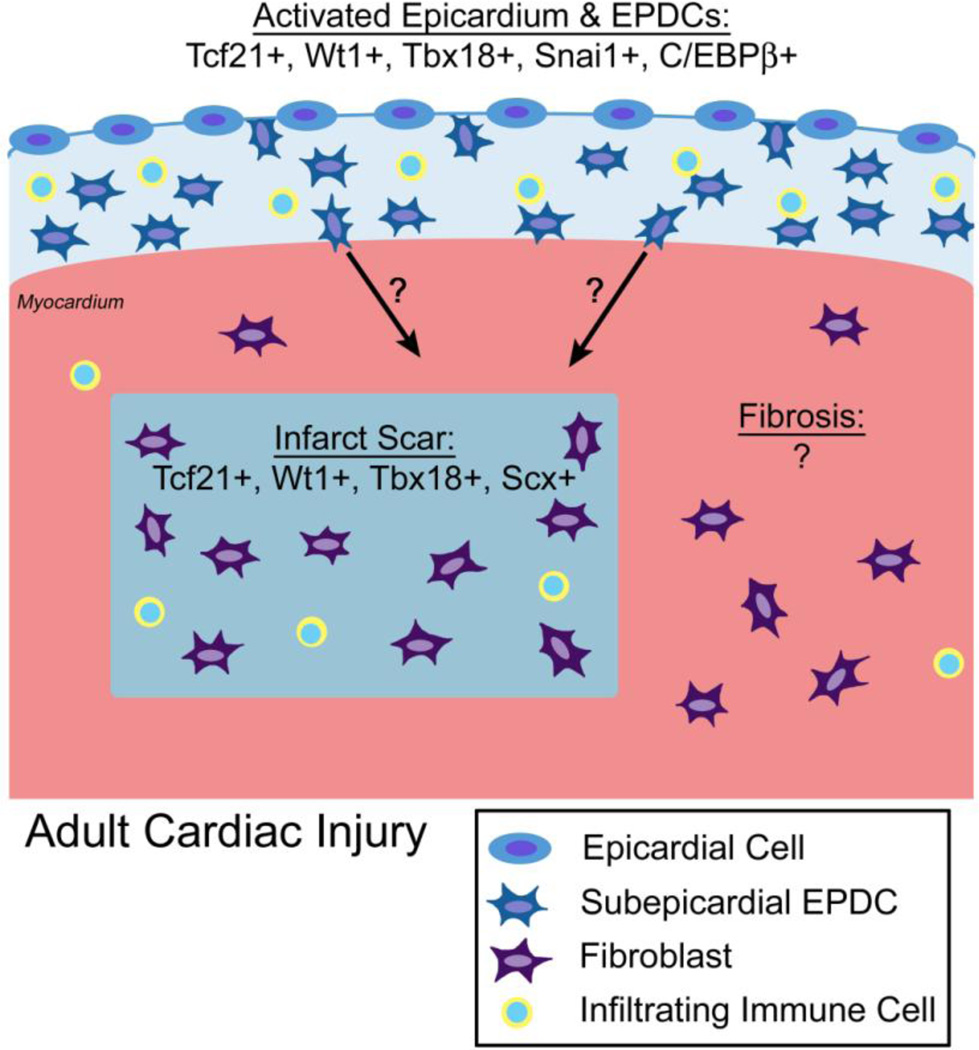
Model depicting epicardial cell reactivation and expression of transcription factors, including Tcf21, Wt1, Tbx18, Snai1, and C/EBPβ, following myocardial infarction (MI) in the adult heart. Activated epicardial cells undergo EMT and invade the subepicardial space following MI. The ultimate fate of activated EPDCs and their ability to invade the myocardium in the infarcted heart has not yet been fully characterized. In the area of the infarct scar Tcf21, Wt1, Tbx18, and Scleraxis (Scx) also are expressed, and immune cells are present in the activated epicardium and fibrotic scar. Currently, it has not been reported whether epicardial transcription factors are activated in other forms of cardiac fibrosis. See text for details and references.

Multiple signaling pathways required for EPDC lineage development are affected with loss of Wt1. Retinoic acid (RA) signaling is required during cardiac morphogenesis [44]. Retinoid X Receptor α, which binds RA in the nucleus, is required during cardiac development, as *Rxrα* null mice are embryonic lethal by E15 with ventricular hypoplasia and delayed formation of the epicardium [45–47]. Wt1-deficient embryos have decreased expression of *Retinaldehyde dehydrogenase-2 (Raldh2)*, a direct downstream target of Wt1, and epicardial EMT is partially rescued by RA supplementation in Wt1-deficient embryos [12,37]. Interestingly, RA induces *Wt1* expression in proepicardial cells and EPDCs in cell culture supporting a feedforward regulatory mechanism [8]. Canonical Wnt/β-Catenin signaling, required for epicardial EMT, ventricular compaction, and formation of the coronary plexus in mouse embryonic hearts, also is downstream of Wt1 [12,48,49]. In Wt1 null embryos, the epicardium fails to undergo EMT and Wnt signaling is reduced [12,48,49]. Therefore, Wt1 is a master regulator upstream of crucial signaling pathways, including Wnt/β-Catenin and RA, in epicardial development. In addition, Wt1, Wnt/β-Catenin, and Raldh2 are reactivated in mouse models of adult heart disease, including MI, ischemia/reperfusion (I/R), and pressure overload (Figure 2) [16,18,31,50].

Initial Wt1Cre-based lineage studies reported that the majority of Wt1-derived cells differentiate into SM, but that some Wt1-derived cells differentiate into cardiomyocytes and endothelial cells [7]. Wt1 lineage-derived cells also contribute to fibroblasts of the annulus fibrosis, interstitial fibroblasts, and AV valve parietal leaflet interstitial cells [24,30]. Very few, if any, endothelial cells are derived from the Wt1 lineage in these analyses [7,24,30]. The report that Wt1 lineage-positive cells become cardiomyocytes, thereby supporting an epicardial origin for cardiac muscle, is controversial [51,52]. Caveats to this approach are that Wt1 expression is not completely epicardial-specific in addition to potential leakiness of Cre expression and inefficiency of recombination inherent to the Wt1Cre mouse lines [51,52]. Tamoxifen-inducible Wt1Cre lines add temporal and spatial specificity, but inefficient and variable recombination following tamoxifen induction is a concern with the Wt1CreERT2 mouse line [51,52]. It remains controversial whether small subpopulations of Wt1 lineage-positive epicardial cells become cardiomyocytes or endothelial cells. However, there is general agreement that the majority of Wt1Cre-positive epicardial derivatives become fibroblasts and vascular SMCs [7,24,51].

### 3.2. Tcf21

The bHLH transcription factor Tcf21 (Pod1/Epicardin/Capsulin) is expressed in developing mesothelial cell populations, including the PE and epicardium, as well as kidney, lung, and reproductive tract [53–55]. Loss of Tcf21 leads to kidney and lung defects, spleen agenesis, and neonatal lethality [56,57]. In the heart, Tcf21 is required for normal epicardial development and regulates EPDC differentiation into SM and fibroblast lineages [8,13]. Tcf21 deficiency leads to aberrant SM differentiation in the subepicardial mesenchyme and a paucity of cardiac fibroblasts in the myocardial interstitium [8]. Expression of Tcf21, like Wt1, is induced by RA signaling in EPDCs, and RA inhibits SM differentiation of PE derivatives [8,58]. Tcf21 expression is downregulated in differentiated vascular SM in the myocardial interstitium, consistent with a repressive role in the differentiation of this lineage. Thus, Tcf21 and RA signaling together inhibit SM gene expression and differentiation in EPDC progenitor cells prior to their localization in the coronary vasculature. In contrast, Tcf21 expression promotes cardiac fibroblast identity and persists in differentiated cardiac interstitial and adventitial fibroblasts in the postnatal and adult heart [8,13,59].

Tcf21 heterodimerizes with the class I bHLH transcription factor E12 [60,61]. Together, Tcf21 and E12 negatively regulate transcription [60,62]. Analysis of *Xenopus* embryos indicates that Tcf21 functions as a transcriptional repressor with other repressor proteins to regulate PE-specific gene expression [63]. Additional bHLH dimerization partners for Tcf21 have not been described, nor have Tcf21 downstream targets been identified in the heart *in vivo*. Studies using a mesenchymal cell line derived from adult mouse kidney determined that Tcf21 binds to E-box DNA consensus sequences (CAnnTG) in the *SM22α*, *Calponin*, and *αSMA* promoters [64]. Overexpression of Tcf21 alone leads to decreased expression of SM22α, Calponin, and αSMA protein, whereas overexpression of Tcf21 and E2A results in increased SM22α, Calponin, and αSMA protein expression [64]. Therefore, expression of *E2A,* which encodes the E12 and E47 transcription factors, may influence the role of Tcf21 in terms of SM and myofibroblast downstream targets [65]. In addition to acting as a transcriptional repressor, Tcf21 contains an activation domain at its C-terminus [66,67]. While expression of SM22α, Calponin, and αSMA is increased in Tcf21 null hearts [8], direct regulatory interactions of Tcf21 with these gene regulatory elements have not yet been established in EPDCs. The dynamic and differential mechanisms by which Tcf21 regulates cell fate have yet to be determined. Likewise, the identity of Tcf21 E-box binding partners is likely to influence Tcf21 function in different contexts [62].

Fate mapping studies with the tamoxifen-inducible Tcf21iCre mouse line demonstrate that Tcf21iCre-derived cells contribute to fibroblasts of the coronary adventitia and myocardial interstitium, in addition to coronary vascular SMCs, when Cre activity is induced during embryogenesis [59]. In addition, Tcf21iCre-derived cells are detected in the gonads, lung, spleen, adrenal gland, and facial skeletal muscles [59]. Interestingly, in the heart, lung, kidney, spleen, adrenal gland, testes, and ovaries, Tcf21iCre-derived cells contribute to interstitial cells that support organ function [59]. Therefore, Tcf21 regulation of interstitial fibroblast formation may be conserved throughout the developing embryo. In the heart, fate mapping of the embryonic Tcf21 lineage marks fibroblasts and SMCs, but not cardiomyocytes or endothelial cells [59,68]. Postnatal induction of Tcf21iCre activity leads to recombination in cardiac interstitial cells, but not endothelial cells, supporting a homeostatic role for Tcf21 in fibroblast lineages after birth [59]. As determined by genome-wide association studies of human coronary artery disease (CAD), a variant of TCF21 is associated with increased risk of CAD in European and Chinese Han populations [69,70]. Likewise, TCF21 is expressed in human cardiac fibrotic disease and ischemic cardiomyopathy ([71]; Braitsch, unpublished). In addition, Tcf21 is reactivated following myocardial injury in adult mouse and zebrafish models (Figure 2) [15,16,18,31,68]. Therefore, Tcf21 is likely to play an important role in adult cardiac homeostasis and disease.

### 3.3. Tbx18

Tbx18, a member of the T-box transcription factor family, is expressed in the PE, epicardium, somites, limb buds, and genital ridge [72,73]. Mice lacking Tbx18 die at birth due to cyanosis resulting from severe defects of the axial skeleton [10,74]. In the heart, Tbx18 contributes to, and is required for, formation of the sinus horn myocardium at the venous pole of the heart [75]. Loss of Tbx18 does not appear to affect epicardial development, as EPDCs are apparently unaffected in the *Tbx18^−/−^* mouse heart [74]. It is possible that Tbx20, which is expressed in the epicardium and subepicardial EPDCs, may have overlapping or redundant functions with Tbx18 in these cells [76]. Lineage-tracing analysis of a Tbx18Cre knock-in allele indicates that cells from the Tbx18Cre lineage differentiate into fibroblasts, vascular SMCs, and cardiomyocytes [3]. However, Tbx18 is actively expressed in myocardium of the interventricular septum and left ventricle during mouse embryogenesis from E10.5–E16.5, supporting a nonepicardial source for Tbx18 lineage-positive cardiomyocytes [75,77]. In contrast, studies by multiple groups confirm that vascular SM and cardiac fibroblasts, but not endothelial cells, arise from a Tbx18-positive epicardial lineage [3,77,78].

T-box transcription factors can act as transcriptional activators and/or repressors [73]. In the developing somites, Tbx18 maintains anterior somite identity by acting as a transcriptional repressor of *Delta-like 1* (*Dll1*), a Notch effector [79]. In EPDCs, there is evidence that Tbx18 functions as a transcriptional repressor of SM differentiation, since ectopic expression of a transcriptional activator Tbx18VP16 leads to premature SM differentiation in the epicardium [10]. Tbx18VP16-mediated SM differentiation in epicardial cells is reversed by Notch inhibition *in vitro* [10]. However, few cardiac-specific downstream targets of Tbx18 have been identified. Tbx18 directly binds and promotes epicardial *Snai2* expression, thereby promoting epicardial EMT in cell culture [40]. Together these studies indicate that Tbx18 maintains progenitor cell identity by acting as a transcriptional repressor during embryonic development, often upstream of Notch signaling. In addition, Tbx18 is reactivated in epicardial cells in adult ischemic heart disease (Figure 2) [16,31].

### 3.4. Nfatc1

Nfatc1 is a member of the nuclear factor of activated T cells family of transcription factors, which are activated and localized to the nucleus by Ca^2+^ signaling via the calcium-responsive phosphatase calcineurin [80]. Loss of Nfatc1 in mice leads to lethality at E12.5–E14.5 with defects in heart valve remodeling [81,82]. Nfatc1 is expressed in the endocardial cushions and remodeling heart valves, as well as in the PE, epicardium, and EPDCs during heart development [9,83,84]. In the developing valves, Nfatc1 is required to promote endocardial cushion proliferation through the VEGF pathway and to regulate heart valve remodeling via Receptor Activator of Nuclear factor Kappa-B Ligand (RANKL) signaling [83,85,86]. In EPDCs, Nfatc1 is required for invasion of the myocardium, and mice deficient in epicardial Nfatc1 have decreased interstitial fibrous matrix deposition and exhibit neonatal lethality [9]. Likewise, epicardial loss of Nfatc1 results in decreased coronary vessel penetration, without affected SM differentiation, in the embryonic mouse heart [9]. Specifically, epicardial Nfatc1 is necessary for RANKL promotion of *CathepsinK (Ctsk)* mediated EPDC invasion of the myocardium [9]. *Ctsk* is an ECM remodeling enzyme that facilitates cell migration and is a transcriptional target of Nfatc1 (Table 1), first defined in osteoclast cell lineages [87,88]. Therefore, Nfatc1 is required for *Ctsk* expression and cell invasion, necessary for EPDC lineage development, in a mechanism that also is active in developing osteoclasts and remodeling heart valves.

Nfatc1 also has been implicated in coronary endothelial lineage development. Nfatc1 is expressed in differentiated coronary endothelial cells, and calcineurin/NFAT signaling is required for coronary angiogenesis during embryonic heart development [9,89]. Targeted deletion of Nfatc1 with Wt1Cre or Gata5Cre does not prevent differentiation of coronary endothelial cells, but these Cre lines are not generally considered to be active in the endothelial lineage [9,51]. However, calcineurin/NFAT signaling is required in endothelial cells for coronary vessel development and is induced by VEGF signaling [89]. Recently, endocardial endothelial cells were reported to be a source of coronary endothelial cells based on restriction of Nfatc1 expression to the endocardium [27]. While these data do not take into account Nfatc1 expression in the epicardium and its necessity for EPDC invasion, they do support multiple sources of coronary endothelial cells that warrant further investigation [6,9,27]. In addition, the specific functions and downstream targets of Nfatc1 in coronary endothelial cell differentiation have not been identified.

### 3.5. Snai1 and Snal2

The zinc finger transcription factors Snai1 (Snail1) and Snai2 (Snail2, Slug) are robustly expressed in the epicardium and EPDCs of mouse and chick embryonic hearts [90,91]. Snai1 promotes EMT in the endocardial cushions as well as in other organ systems and during tumorigenesis, in part via the Snai1 downstream target *Mmp15* [92,93]. However, there is conflicting evidence for the requirement for Snai1 and Snai2 in epicardial EMT [39,90,94]. In cultured avian epicardial cells, Snai1 overexpression promotes cell migration and invasion [94]. Similarly in mouse epicardial cell cultures, loss of Snai2 inhibits EMT, and *Snai2* gene expression is dependent on Wt1 and Tbx18 [40]. Deletion of Wt1 from the more broadly expressed Gata5Cre lineage leads to loss of *Snai1* expression with concomitant epicardial EMT defects with embryonic lethality [39,95]. In contrast, *in vivo* loss of Snai1 in Wt1Cre or Tbx18Cre lineages does not affect epicardial EMT or differentiation [90]. In a variety of cell types, Snai1 represses expression of *E-cadherin* and other adhesion molecules, which are required to maintain epithelial integrity [96,97]. Wt1Cre-mediated loss of the Notch pathway transcriptional activator *Rbpj* leads to decreased expression of *Snai1*, consistent with an observed EMT defect, as well as aberrant coronary SM differentiation [98]. Together these studies suggest that Notch signaling regulates Snai1 and E-cadherin, both of which affect epicardial EMT. This same regulatory hierarchy also is active in endocardial cushion EMT [99]. In addition, *Snai1* expression is reactivated in the infarct scar following MI (Figure 2) [31].

### 3.6. Twist1 and Hand2

In addition to Tcf21, the bHLH transcription factors Twist1 and Hand2 also have been implicated in EPDC development. Twist1 is expressed in EPDCs of avian embryos at the same time it is expressed in endocardial cushions, where it promotes mesenchymal cell proliferation and migration [76]. In mice, EPDCs isolated from mouse AV canals express Twist1, in addition to Snai1, Snai2, and Smad1 markers of EMT [30]. However, a specific function for Twist1 in the epicardium or EPDCs has not yet been demonstrated. In endocardial cushions, Twist1 promotes expression of genes associated with cell proliferation and migration, and a similar regulatory mechanism may be active in EPDCs [76,100]. For example, *Tbx20* is expressed in EPDCs as well as endocardial cushions and is a direct downstream target of Twist1 in endocardial cushion cells [76,100]. Likewise, Hand1 is expressed at the venous pole of the heart and cells of the Hand1 lineage contribute to epicardial progenitors [101]. In addition, loss of Hand2 in the Hand1 lineage leads to epicardial blistering, abnormal coronary vessel development, and loss of cardiac fibroblasts [101]. Hand2 promotes expression of *Pdgfra,* which is required for epicardial EMT and epicardium-derived cardiac fibroblasts [101,102]. Additional studies are necessary to define the specific functions and transcriptional targets of Twist1, Hand1, and Hand2 in epicardial lineage development.

### 3.7. Scleraxis and Sox9

Scleraxis (Scx) is a bHLH transcription factor originally reported to be important in tendon development, and it also functions in cell lineage diversification in heart valvulogenesis [103–105]. Scx is expressed in a subdomain of the mouse PE, beginning at E9.5, and in the epicardium at E10.5 [6]. In the PE, cells that express Scx do not express Wt1 or Tbx18, demonstrating heterogeneity of this progenitor population [6]. ScxCre-derived cells contribute to coronary endothelial cells on the surface of the heart and also to cardiomyocytes in the LV. However, ScxCre-positive cells are rarely detected in SM at E12.5, in contrast to Wt1Cre or Tbx18Cre-derived cells, providing evidence for distinct compartments of proepicardial cells that give rise to endothelial versus fibroblast and SM lineages. The specific function(s) of Scx in epicardial development has not been demonstrated, although loss of Scx leads to persistent expression of EMT markers and heart valve remodeling defects at E17.5 in mice [106]. Interestingly, in adult *Scx^−/−^* mice, thickening and increased collagen deposition are apparent in the AV annulus and mitral valve parietal leaflet that are derived from epicardium [24,30,106]. In adult cardiac fibroblasts, Scx directly regulates *Col1a2* gene expression, and Scx expression also is induced after MI, supporting a role in cardiac fibrosis (Figure 2) [107]. However, additional studies are necessary to define the specific functions of Scx in epicardium-derived cell lineage development or pathogenesis related to EPDCs.

Sox9 is an SRY-related transcription factor that is crucial for heart valve development [104]. During valvulogenesis, Sox9 is required for endocardial cushion EMT, progenitor cell proliferation, and proteoglycan-rich cell lineage development [104,108]. Sox9 also is expressed in EPDCs and is sufficient to promote epicardial EMT and migration [102]. Therefore, mechanisms regulating EMT and mesenchymal proliferation may be conserved in endocardial cushions and epicardium. However, little is known of Sox9 functions in EPDCs, and defective EPDC lineage development has not been reported in Sox9-deficient mouse embryos.

### 3.8. C/EBP

In a recent report, CCAAT/enhancer binding proteins (C/EBPs) were identified as critical transcriptional regulators of epicardial gene expression during development, that are also activated after cardiac injury [32]. Analysis of conserved enhancer regions of *Raldh2* and *Wt1* revealed the presence of multiple C/EBP binding sites required for epicardial expression of both genes during embryonic development. In adult mice, epicardial C/EBPβ expression is activated with cardiac ischemic injury (Figure 2), and C/EBP function is required for epicardial *Wt1* and *Raldh2* gene activation. Loss of C/EBP function with cardiac ischemic injury leads to reduced fibrosis, decreased inflammation, and improved cardiac function. C/EBPs likely act with other epicardially expressed transcription factors in yet unidentified regulatory hierarchies in EPDC development and response to cardiac injury.

## 4. Transcriptional Regulation of EPDC Lineages in Adult Cardiac Regeneration, Injury, and Fibrosis

Adult zebrafish, unlike adult mammals, have the ability to regenerate cardiac muscle after resection or cryoinjury [109,110]. Epicardial activation, evident in increased Raldh2, Wt1, Tbx18, and Tcf21 expression and increased cellularity of the epicardium, occurs with injury in adult zebrafish [68,111,112]. However, the new muscle arises from existing cardiomyocytes during the regenerative process [113,114]. Fate mapping of Tcf21-positive epicardial cells demonstrates that they contribute to perivascular cells, but not cardiomyocytes, during regeneration [68]. Epicardial contributions to the regenerate were not observed, although *Raldh2* expression was increased, indicative of epicardial activation with injury [111]. The role of epicardial activation and specific functions of transcription factors in the activated epicardium are not known; however, RA signaling emanating from the epicardium and endocardium is required for regeneration [111]. Since *Raldh2* is a downstream target of Wt1 in mouse embryo EPDCs, a similar regulatory interaction may be conserved in zebrafish regeneration. In mice, neonates can renegerate myocardium after ventricular resection, but this ability is lost by postnatal day 7 [115]. Wt1 and *Raldh2* expression is increased in the neonatal mouse epicardium after injury, but proliferation of existing cardiomyocytes was observed to be the source of regenerated myocardium. Together, studies in zebrafish and neonatal mice demonstrate a potential indirect inductive role for EPDC activation in cardiac regeneration and revascularization, but do not support direct EPDC contributions to new cardiomyocyte populations.

In adult humans and mice, EPDC transcriptional programs are reactivated with cardiac injury and fibrosis. Epicardial expression of Wt1, Tcf21, Tbx18, and Raldh2 is increased after MI in mice and in human diseased hearts [16,18,31,71,116]. In addition, increased numbers of cells, that include EPDCs and infiltrating immune cells, are present in the subepicardial space (Figure 2) [18]. Indicators of EMT, including Wnt/β-catenin signaling, Notch signaling, and Snai1 expression, are induced, consistent with reactivation of epicardial cells and generation of new EPDCs of unknown fate or function [18,31,50,71,117]. Fate mapping studies of the tamoxifen-inducible Wt1CreER lineage demonstrated that the activated EPDCs that express Wt1 become fibroblasts and SM cells, but not cardiomyocytes or endothelial cells, after MI in mice [18]. Increased EPDC expression of proangiogenic factors also was observed in these studies, but it is not clear if these genes are directly regulated by EPDC transcription factors such as Wt1, Tcf21, or Tbx18 that also are induced with MI [18]. In addition to subepicardial cells and EPDCs, Wt1, Tcf21, and Tbx18 also are expressed in interstitial and perivascular fibrotic regions of human and mouse diseased heart, but the functions of these factors in cardiac fibrosis and origins of these cells have not yet been determined ([18,31]; Braitsch, unpublished). While it is clear that transcription factors expressed in embryonic EPDCs also are expressed in the adult epicardium with cardiac injury and fibrotic regions of diseased hearts, additional research is necessary to determine their specific regulatory mechanisms and potential therapeutic applications in human cardiovascular disease.

## 5. Conclusions and Future Perspectives

Since the initial reports of EPDCs in the 1990s, there have been rapid discoveries of transcription factors and signaling pathways important for epicardium-derived cell lineage development. More recently, epicardial transcription factor expression has been reported in adult cardiac disease. While EPDC transcription factors have been used as markers for progenitor cells and epicardial activation, specific information related to transcriptional targets and cell lineage regulation is limited. Much is yet to be learned in terms of transcriptional regulatory networks and lineage determination mechanisms in the developing epicardium and its derivatives. Interestingly, epicardial transcription factors, including Wt1, Tcf21, and Tbx18, also are expressed in a variety of mesothelial progenitor lineages, and it is likely that they have similar roles in fibroblast and SM development in multiple organs. Intersection with Notch, RA, and Wnt signaling pathways also may be conserved in the development of these lineages. Transcription factors expressed in the epicardium prior to or during the generation of EPDCs are in many cases also expressed once EPDCs reach their final destination in the heart and differentiate into fibroblasts and SMCs. Thus, it seems likely that there will be more than one function for these factors depending on timing (epicardium versus EPDC) and environment (surface, myocardial interstitium, coronary vessel). Even less is known of activated EPDC function and transcriptional regulatory mechanisms in adult cardiovascular disease. There has been much recent excitement and high impact research in this area, but specific pathologic or reparative functions of EPDCs and associated transcription factors are yet to be fully defined. While it is possible that EPDCs contribute to cardiac repair, especially in the promotion of vascularization, it seems very likely that EPDCs also contribute to pathological fibrosis and, potentially, heart failure. Thus, efforts directed towards harnessing EPDCs in the treatment of human cardiovascular disease should proceed with caution.

## Figures and Tables

**Table 1:** Transcription factor expression and function in epicardial development (see text for details and references).

Gene	Loss-of-function cardiac phenotype a	Known downstream targets expressed in EPDCs	References
Wt1	Ventricular non-compaction; impaired epicardial EMT; impaired coronary plexus formation; pericardial hemorrhaging; die by E13.5	*Itga4, Nestin, TrkB,* *Coronin1B, Raldh2, Snai1,* *Snai2*	[11,12,37–43]
Tbx18	Caval vein defects; sinus horn myocardial hypoplasia; neonatal lethality	*Snai2*	[40,74,75]
Tcf21	Aberrant smooth muscle differentiation; loss of cardiac fibroblasts; pericardial hemorrhaging; neonatal lethality	None identified	[8,13,57]
Nfatc1	bReduced cardiac fibrous matrix with decreased coronary vessel penetration; neonatal lethality	*Ctsk*	[9,87,88]
Snai1	b,cPhenotypically normal and viable	*E-cadherin, Mmp15*	[92,93,96,97]
Snai2	Phenotypically normal and viable	None identified	[40]
Sox9	Hypoplastic endocardial cushions. Embryonic lethality at E11.5–E12 due to congestive heart failure.	None identified	[102,104,108]
Scleraxis	Thickened valves; viable	*Col1a2*	[106,107]
C/EBP	dImproved cardiac function after ischemia/reperfusion injury	*Raldh2, Wt1*	[32]
Hand2	eEpicardial blistering; abnormal coronary vessel development; loss of cardiac fibroblasts; persistent truncus arteriosus. Embryonic lethality by E14.5.	*Pdgfra*	[101]
Twist1	Abnormal outflow tract endocardial cushion mesenchyme. Embryonic lethality by E11.5.	*Tbx20, Snai2*	[30,76,100]

a Described phenotypes are due to knockout mouse models, except in cases of epicardial-specific gene deletion, as indicated; Gene (floxed allele) was deleted from the

b Wt1Cre,

c Tbx18Cre, or

e Hand1Cre lineages, as indicated;

d Antisense adenoviral-mediated knockdown.
